# The Gut Microbiome on a Periodized Low-Protein Diet Is Associated With Improved Metabolic Health

**DOI:** 10.3389/fmicb.2019.00709

**Published:** 2019-04-04

**Authors:** Zhencheng Li, Torben Sølbeck Rasmussen, Mette Line Rasmussen, Jingwen Li, Carlos Henríquez Olguín, Witold Kot, Dennis Sandris Nielsen, Thomas Elbenhardt Jensen

**Affiliations:** ^1^Section of Molecular Physiology, Department of Nutrition, Exercise and Sports, University of Copenhagen, Copenhagen, Denmark; ^2^Microbiology and Fermentation, Department of Food Science, University of Copenhagen, Frederiksberg, Denmark; ^3^Department of Environmental Sciences, Aarhus University, Roskilde, Denmark

**Keywords:** microbiome, exercise, low protein diet, periodized, intermittent

## Abstract

A periodized (14 days on/14 days off) 5% low protein-high carbohydrate (pLPHC) diet protects against weight gain, improves glucose tolerance in mice and interacts with concurrent voluntary activity wheel training on several parameters including weight maintenance and liver FGF21 secretion. The gut microbiome (GM) responds to both diet and exercise and may influence host metabolism. This study compared the cecal GM after a 13.5-week intervention study in mice on a variety of dietary interventions ± concurrent voluntary exercise training in activity wheels. The diets included chronic chow diet, LPHC diet, 40 E% high protein-low carbohydrate (HPLC) diet, an obesigenic chronic high-fat diet (HFD) and the pLPHC diet. Our hypothesis was that the GM changes with pLPHC diet would generally reflect the improved metabolic health of the host and interact with concurrent exercise training. The GM analyses revealed greater abundance phylum Bacteroidetes and the genus *Akkermansia* on chronic and periodized LPHC and higher abundance of *Oscillospira* and *Oscillibacter* on HFD. The differences in diet-induced GM correlated strongly with the differences in a range of host metabolic health-measures. In contrast, no significant effect of concurrent exercise training was observed. In conclusion, pLPHC diet elicits substantial changes in the GM. In contrast, only subtle and non-significant effects of concurrent activity wheel exercise were observed. The pLPHC-associated microbiome may contribute to the healthier host phenotype observed in these mice.

## Introduction

Overweight defined as body mass index (BMI) above 25 kg/m^2^ has been suggested to account for about 65–80% of new cases of type 2 diabetes (Kahn et al., 2006). The microbiome of the gastro-intestinal tract has been found to consist of 1100 to 2000 taxa of bacteria in healthy humans (Rajilic-Stojanovic and de Vos, 2014). Gut microbiome (GM) dysbiosis is causally linked to development of obesity and may affect mammalian host metabolism to influence the manifestation and severity of a range of metabolic diseases including insulin resistance and type 2 diabetes (Khan et al., 2014).

Diet is a major environmental factor influencing both the GM and its host (Khan et al., 2014). Obese compared to lean humans in general display decreased alpha diversity, i.e., a decreased richness of bacterial species with lowered gene count (Turnbaugh et al., 2009; Cotillard et al., 2013). In rodent models, a chronic high-fat diet (HFD) is obesigenic and impairs whole-body insulin sensitivity in multiple organs including liver, white adipose tissue, and skeletal muscle (Kleinert et al., 2018). Interestingly, this dietary regimen decreases the diversity of the GM and promotes overgrowth of a range of opportunistic pathogens (Khan et al., 2014). This is proposed to drive HFD-associated pathologies by increasing gut permeability and promoting low-grade inflammation and metabolic disturbances as evidenced by co-housing, antibiotics and fecal transplant studies in mice (Khan et al., 2014; Murphy et al., 2015).

Apart from dietary fat, the other dietary composition macronutrients, carbohydrate and protein, also markedly influences whole-body insulin sensitivity in mice, with a high carbohydrate/low protein diet markedly increasing, and a low carbohydrate/high protein diet decreasing, insulin sensitivity (Newgard et al., 2009; Solon-Biet et al., 2014, 2015). Interestingly, carbohydrate and protein are the major macronutrient components of non-digested food serving as fermentation substrates in the gut, and have been proposed to release a number of fermentation products to influence host insulin sensitivity (Khan et al., 2014). A recent study comparing the effect of 25 diets differing in their macronutrient composition in mice found that changes in the obesity-associated microbiome was dictated by carbohydrate and nitrogen availability, rather than dietary fat content (Holmes et al., 2017). The health-span linked Bacteroidetes/Firmicutes (B/F) -ratio was inversely associated with total protein intake (Holmes et al., 2017), although the subtaxa of these phyla displayed dissimilar responses to the diets. Overall, this suggests that low dietary protein intake causes beneficial changes in the GM which might explain some of the health- and life-span extending benefits of such diets (Laeger et al., 2014; Solon-Biet et al., 2014, 2015; Fontana et al., 2016; Maida et al., 2016).

Exercise training is another environmental factor known to influence whole-body insulin-sensitivity by, e.g., increasing insulin-dependent and independent skeletal muscle glucose uptake (Sylow et al., 2016). Interestingly, different exercise training regimens in mice also has a pronounced influence on the GM, protecting against HFD induced decreases in microbiome diversity (Denou et al., 2016) and increasing certain taxa including *Butyricimonas*, *Prevotella*, and *Akkermansia muciniphila* (Everard et al., 2013; Lamoureux et al., 2017; Liu et al., 2017).

In a recent study, we investigated the impact of a periodized 14 days low protein/high carbohydrate (pLPHC) diet/14 days HFD cycles provided for 3 months in conjunction with voluntary running wheel exercise training (termed exercise training from hereon) on obesity-development, whole-body metabolism, insulin sensitivity, and cell signaling in liver and skeletal muscle (Li et al., 2018a). Our control diets included chow, 60% HFD, LPHC diet, and HPLC diet given chronically for 3 months (Li et al., 2018b). Apart from examining the effects on host metabolism, we collected cecum to perform 16S ribosomal RNA gene amplicon sequencing to explore the interactions between diet and exercise on the GM and correlate these findings with our data on metabolic regulation in these mice. Our overall hypothesis was that obesity-associated changes in the GM would be counteracted by exercise training and pLPHC diet in a synergistic manner.

## Materials and Methods

### Study Design

The overall study design, methods, diet descriptions and a comprehensive metabolic characterization has been published previously (Li et al., 2018a,b). All experiments were pre-approved by the Danish Animal Experimental Inspectorate and complied with the “European Convention for the Protection of Vertebrate Animals Used for Experiments and Other Scientific Purposes.” In brief, retired female C57BL/6J (Janvier, France) breeder mice aged 8–9 months were randomized to either chronic chow diet, low-protein/high-carbohydrate diet, high-protein/low-carbohydrate diet, HFD or a periodized low-protein/high-carbohydrate diet regimen, switching between pLPHC diet and HFD every 14 days. The chow [SF14-162, 63/19/18 E% Carbohydrate(c)/protein(p)/fat(f)], LPHC (SF09-048, 74/5/21 E% c/p/f), and HPLC diets (SF09-069, 29/40/31 E% c/p/f), all from Speciality Feeds, Australia are described in Supplementary Table S1 in Li et al. (2018b). The high fat diet provided 20/20/60 E% c/p/f (Cat.: D12492) was from Research Diet Inc.

At the end of the study, the mice were killed following 2–3 h fasting from 9 a.m. to collect tissues and plasma. Approximately 200 mg of cecum content was collected from a cut made on apex ceci and was then snap frozen in liquid nitrogen until future analysis.

Worth noting, our study did not include a pre-intervention control measurements of cecal microbiome to validate the randomization. Previous studies indicate that this is not a concern if properly randomized (Anhe et al., 2015; Le et al., 2018; Montonye et al., 2018). However, since we in principle cannot know if the GM changed due to the intervention, we have worded the descriptions and conclusions throughout to indicate rather than show changes over time.

### Bacterial DNA Extraction and Sequencing

Approximately 50–120 mg of cecal content was from each sample weighed into Powersoil tubes on a Radwag model AS 220.R2 (Bracka, Poland). DNA was extracted with DNeasy PowerSoil Kit (Cat. No. 12888-100, Qiagen, Germany) by following manufactures instructions, however, the volume of elution buffer was decreased from 100 to 40 μL to increase DNA concentration of the isolates. FastPrep-24 (MP Biomedicals, United States) was applied for the physically disruption of bacterial cells, and Hermle Z216MK (Hermle, Germany) for all centrifugation steps. DNA concentration and purity were assessed using a NanoDrop 1000 spectrophotometer (Thermo Fisher Scientific, United States). DNA samples were subjected to tag-encoded 16S rRNA gene NextSeq-based high throughput sequencing (Illumina, United States). The V3 region (∼190 bp) of the 16S rRNA gene was amplified using primers compatible with Nextera Index Kit (Illumina): NXt_388_F: 5′- TCGTCGGCAG CGTCAGATGT GTATAAGAGA CAGACWCCTA CGGGWGGCAG CAG -3′ and NXt_518_R: 5′- GTCTCGTGGGC TCGGAGATGTG TATAAGAGAC AGATTACCGC GGCTGCTGG -3′ (Integrated DNA Technologies; Leuven, Belgium). PCR reactions containing 12 μl AccuPrimeTM SuperMix II (Life Technologies, CA, United States), 0.5 μl of each primer (10 μM), 5 μl of normalized genomic DNA (∼20 ng/μl), and 2 μl nuclease-free water to a total volume of 20 μl were run on a SureCycler 8800. Cycling conditions applied were: 95°C for 2 min; 33 cycles of 95°C for 15 s, 55°C for 15 s, and 68°C for 30 s; followed by final step at 68°C for 5 min. Agarose gel electrophoresis was performed to ensure that a ∼250 bp PCR product was obtained for each sample, except negative control. To incorporate primers with adapters and indexes, PCR reactions contained 12 μl Phusion High-Fidelity PCR Master Mix (Thermo Fisher Scientific, United States), 2 μl corresponding P5 and P7 primer (Nextera Index Kit), 2 μl PCR product and nuclease-free water for a total volume of 25 μl. Cycling conditions were: 98°C for 1 min; 13 cycles of 98°C for 10 s, 55°C for 20 s and 72°C for 20 s; and 72°C for 5 min. The amplified fragments with adapters and tags were purified using AMPure XP beads (Beckman Coulter Genomic, United States). Pooling and purification were performed with Biomek 4000 pipetting robot (Beckman Coulter). Prior to library pooling clean constructs were quantified using a Qubit Fluorometer (Invitrogen, United States) and mixed in approximately equal concentrations to ensure even representation of reads per sample. Each sample was represented with approximately 40 ng DNA. Subsequently 150 bp pair-ended NextSeq (Illumina, United States) sequencing was performed according to the instructions of the manufacturer.

### Olink Exploratory Mouse Panel

Plasma samples collected within 5 min post-mortem were shipped to Olink (Uppsala, Sweden) for analyses on their Mouse exploratory panel, using single measurements on 1 μl of plasma. A full list of the 92 proteins on this panel is available at https://www.olink.com.

### Data Processing and Analysis

Raw NextSeq reads were processed by the IMNGS (Lagkouvardos et al., 2016) pipeline (version 1.0 Build 1808) which is based on UPARSE (Edgar, 2013), and USEARCH (Edgar, 2010). Allowed mismatches was set to 2, minimum fastq quality score for trimming of unpaired reads to 3, max number of expected errors in paired sequences to 3, and the minimum relative OTU abundance to 0.25%. The analysis delivered 9,012,489 quality and chimera-checked reads (average 219,816 ± 72,910 reads per sample). OTU’s were clustered at 97% sequence identity. Alignment of IMNGS generated OTU-sequences with SINA version 1.2.11 (Pruesse et al., 2012) (default settings) was performed to obtain the highest possible level of taxonomy of each OTU. The R-based RHEA pipeline (Lagkouvardos et al., 2017) (version 1.1.1.) were used for data analysis yielding rarefaction curves, correlations, group significance (taxonomy and meta-data), and taxonomic binning. Bray–Curtis and Weighted UniFrac (Lozupone and Knight, 2005) represented the phylogenetic beta-diversity analysis and the Shannon index determinations were used for alpha-diversity analysis (see Supplementary Figure S1 for alpha-diversity: Faith phylogenetic diversity, Richness. Beta-diversity: weighted and unweighted Unifrac). Normalization of sequencing depth was done by rarefaction based on 74583 reads via division by the sum of sequences in a given sample and multiplication by the minimum sum across all samples. See Supplementary Figure S3 for rarefaction curves for each individual sample. Data files processed by RHEA were imported to Quantitative Insight Into Microbial Ecology 2 (Caporaso et al., 2010) (2018.4 build 1525276946) for visualization and for group significance of the alpha and-beta-diversity. QIIME 2 is an open source software package for Oracle Virtual Box (Version 5.2.10). The non-parametric Kruskal–Wallis and ANOSIM were used to calculate group significance for, respectively, alpha- and beta-diversity. Taxonomic heatmap was generated with QIIME 1.9.17 (make_otu_heatmap.py) based on binned taxonomy table from RHEA. The volcano plots in Figure 3 were made using OriginPro 2017 with the cut-off set to *p* < 0.05 and fold change >1.5. The *p*-values used were computed using Student’s *t*-test in Perseus 1.6.1.3 (Tyanova et al., 2016).

## Results

### HFD Seems to Lower and pLPHC Diet Increase Alpha-Diversity but Less So Than Chronic LPHC Diet

**Figure 1:**
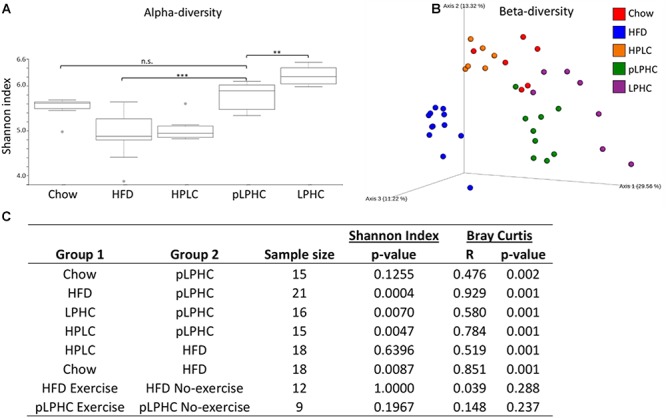
**(A)** Alpha-diversity measured as Shannon index. Gray dots are outliers. **(B)** PCoA plot of the beta-diversity based on the Bray-Curtis metric. **(C)** Statistical pairwise group comparisons. HFD, High fat diet; HPLC, High protein low carbohydrate; pLPHC, periodized low protein high carbohydrate. ^∗^*p* < 0.05, ^∗∗^*p* < 0.0071, ^∗∗∗^*p* < 0.0005.

Initially, we compared the effects of our different 13.5-week diet and diet+exercise training interventions on the GM alpha-diversity in the cecum. Consistent with previous findings, we found that HFD diet mice displayed lower Shannon-diversity compared to chow diet (Figure 1A). Conversely, mice on the pLPHC diet regimen exhibited significantly (*p* = 0.0004) higher Shannon-diversity compared to HFD (Figure 1A). Chronic LPHC diet had a more pronounced additional effect on Shannon-diversity compared to pLPHC diet. The HPLC diet resembled the HFD group (*p* = 0.64) and was significantly different from the other groups (*p* < 0.025). Similar observations were made for the number of the bacterial Richness and Faith phylogenetic diversity in Supplementary Figure S1.

### Beta-Diversity Seems to Be Influenced by All Diets but Not Exercise Training

For beta-diversity, i.e., the difference in the taxonomic abundance profiles between our interventions, a principal coordinate analysis (PCoA) clearly indicated that the pLPHC groups were pushed in the direction of the chow and chronic LPHC diet groups (Figure 1B). Overall, our statistical analyses revealed a significant (*p* ≤ 0.002) separation between the intervention groups for chow vs. HFD, chow vs. pLPHC diet, HFD vs. pLPHC diet, and pLPHC vs. chronic LPHC diet (Figure 1B and Supplementary Figure S1). Of particular relevance, the pLPHC diet group was significantly and strongly separated from the HFD group (*R* = 0.929, *p* = 0.001) and separated from the chow (*R* = 0.476, *p* = 0.001) and chronic LPHC group (*R* = 0.580, *p* = 0.001). Thus, the higher *R*-value supported the PCoA analysis of a greater separation of pLPHC vs. HFD than pLPHC vs. chow and chronic LPHC. HPLC was significantly different from the HFD (*R* = 0.519, *p* = 0.001) and pLPHC (*R* = 0.784, *p* = 0.001) with regards to beta-diversity (Figure 1C). In contrast, no effect of exercise training on either alpha or beta-diversity measures were observed (Figure 1C and Supplementary Figure S1). Additional weighted and unweighted UniFrac metrices were applied and produced the same overall tendencies (see Supplementary Figure S1). Thus, the bacterial composition of the gut seems strongly influenced by diet and a pLPHC diet regimen seems sufficient to significantly change this parameter.

### pLPHC Diet Seems to Push the Bacterial Composition Toward a Health-Associated Phenotype

**Figure 2:**
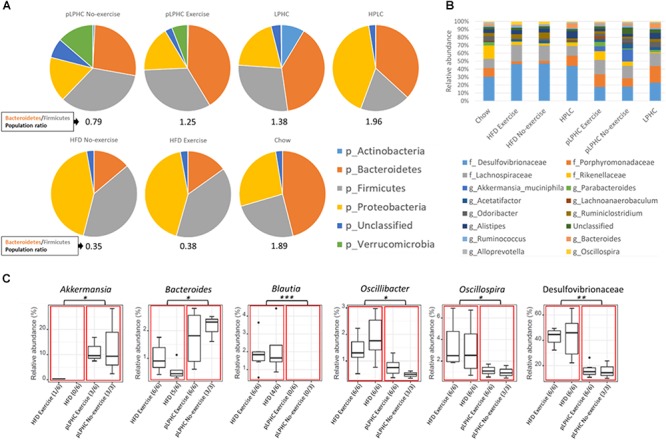
**(A)** Pie charts showing the relative taxonomic abundance of bacterial phyla as determined by 16S rRNA gene amplicon sequencing. **(B)** Bar chart of the 16 most abundant bacterial families and genera. **(C)** Group significance analysis of selected bacteria suggested in the literature to have a positive or negative impact on metabolic diseases. The red boxes divide the chart into a HFD and pLPHC and the black dots are outliers. HFD, high fat diet; HPLC, high protein low carbohydrate; pLPHC, periodized low protein high carbohydrate. ^∗^*p* < 0.05, ^∗∗^*p* < 0.005, ^∗∗∗^*p* < 0.0005.

At the phylum level, the B/F ratio (Figure 2A) was significantly (*p* = 0.0009) lower in the HFD group compared to chow (0.35 and 1.89, respectively). Both pLPHC and chronic LPHC diet had B/F ratios in-between the chow and HFD groups (0.79 and 1.38, respectively). The HPLC diet had a slightly elevated B/F-ratio compared to chow at 1.96. Concurrent exercise training caused a numerically higher (non-significant) difference in B/F ratio in the pLPHC groups (no-exercise: 0.79 vs. exercise: 1.25) compared to the HFD groups (no-exercise: 0.35 vs. exercise: 0.38). The prevalence of the phylum of Proteobacteria seemed increased (*p* < 0.05) in the HPLC and HFD group compared to any other group (Figure 2A).

The overall genus level changes are illustrated in Figure 2B. Significant differences between HFD vs. pLPHC diet were observed, with higher *Akkermansia* and *Bacteroides* in the pLPHC groups and lower abundance of *Blautia*, *Oscillibacter*, *Oscillospira*, and Desulfovibrionaceae in the pLPHC groups (Figure 2C and Supplementary Figure S2). No significant effects of concurrent exercise were found. Thus, pLPHC diet appears sufficient to elicit changes in the bacterial composition of the gut which have previously been associated with health-benefits to the host.

### A Targeted Multiplex Analysis of Plasma Proteins Revealed pLPHC Diet and Exercise-Induced Differences in Multiple Proteins

**Figure 3:**
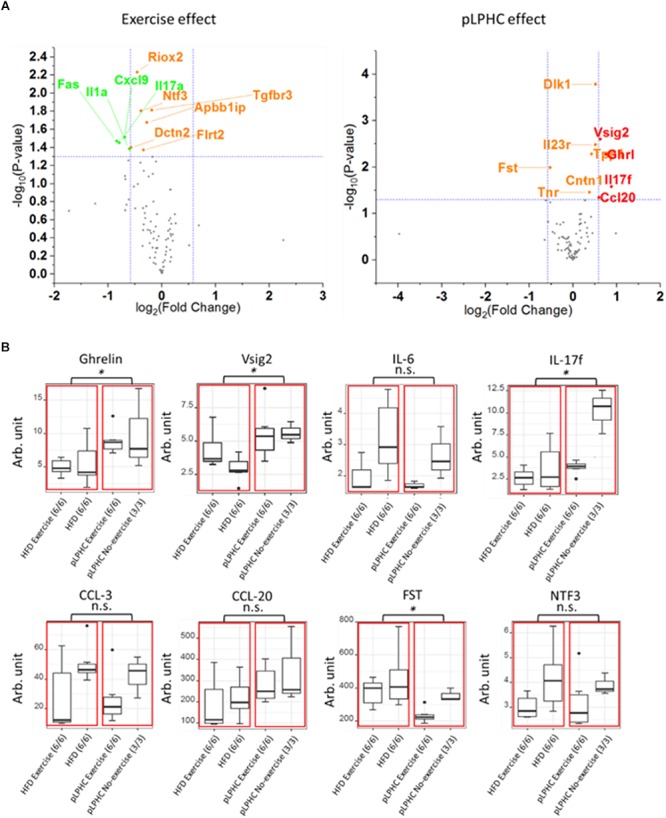
**(A)** Volcano plot illustrating the effect of exercise or pLPHC (*X*-axis, log2 scale, cut-off >1.5 fold) vs. statistical significance (*Y*-axis, log10 scale, cut-off *p* < 0.05) on the expression of metabolic and inflammation related proteins in plasma measured using the Olink Exploratory mouse panel. Stippled lines denote cut-off values. Red – increase by pLPHC diet vs. HFD; Green – decreased by exercise vs. non-exercise. **(B)** Box and whiskers plots showing protein expression of eight significantly different plasma proteins (Ghrelin, Vsig2, IL-6, IL-17f, CCL-3, CCL-20, FST, and NTF3). The red boxes marks statistical analysis of the HFD and pLPHC group, and the black dots are outliers. HFD, high fat diet; HPLC, high protein low carbohydrate; pLPHC, periodized low protein high carbohydrate. ^∗^*p* < 0.05, n.s. – not significant.

To test the influence of pLPHC diet and exercise training on plasma proteins, particularly immune function-related proteins, we applied an exploratory 92 mouse protein multiplex biomarker panel to plasma samples from our different intervention groups. We detected 89 of 92 proteins in our plasma samples and these are shown as volcano plots divided by either exercise or diet in Figure 3A,B, with Supplementary Table S1 listing the 19 significantly different proteins found. Since we measured plasma, we chose to focus only on known secreted proteins (Supplementary Table S2). This resulted in deregulation of a total of 8 known plasma proteins including a number of immune function biomarkers, specifically higher Interleukin 17f (IL-17f) with pLPHC diet vs. HFD and lower IL-17f with exercise vs. non-exercise, higher V-Set And Immunoglobulin Domain Containing 2 (Vsig2), and Chemokine (C-C motif) ligand 20 (Ccl20) with pLPHC diet vs. HFD, and lower Interleukin 6 (IL-6) and Chemokine (C-C motif) ligand 3 (Ccl3) with exercise vs. non-exercise. In addition, Follistatin (Fst) was lower and Ghrelin (Ghrl) higher on pLPHC diet vs. HFD, whereas Neurotrophin 3 (Ntf3) decreased significantly with exercise vs. non-exercise. The significant proteins from Supplementary Table S2 are presented as bar graphs in Figure 3B. Overall, pLPHC diet tended and exercise training appeared to significantly lower the classical proinflammatory cytokine IL-6. In addition, these interventions significantly seemed to change a number of other proteins of potential relevance to the pLPHC phenotype.

### The pLPHC Diet Induced Changes in GM Are Associated With Obesity-Protection, Improved Insulin Sensitivity and Decreased Inflammation

**Figure 4:**
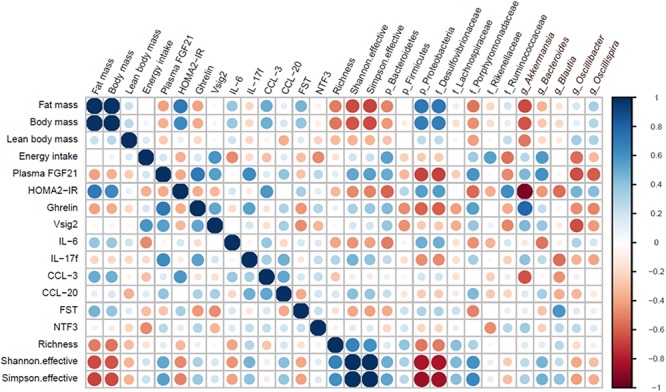
Heatmap illustrating positive (blue) or negative (red) correlations between selected bacteria and physiological host parameters.

Next, we compared our current analysis of the GM to our previous published meta-data on a number of health-related parameters in the same mice (Li et al., 2018a). To briefly sum up our published findings, pLPHC diet mice vs. HFD controls showed improved whole-body glucose tolerance, HOMA2-IR, increased total energy intake but also increased adaptive thermogenesis leading to overall reduced feed efficiency and weight gain-protection, resulting from significant loss of fat mass and tendency to loss of lean body mass. Concurrent running wheel access blunted pLPHC-induced FGF21 secretion and tended to decrease both the body and fat mass gain on HFD and loss on pLPHC diet. These correlations are summed up as a heatmap in Figure 4 with selected statistically significant individual Pearson correlations shown in Supplementary Table S3. As expected, fat mass correlated tightly and positively with total body mass, lean body mass and insulin-resistance (HOMA2-IR) (Figure 4). Fat mass also tended to show a positive correlation with the proinflammatory cytokine IL-6 (*r* = 0.39, *p* = 0.15), the chemokine CCL-3 (*r* = 0.49, *p* = 0.023) and an inverse correlation (*r* = -0.41, *p* = 0.067) with plasma FGF21 (Figure 4). This is consistent with HFD increasing body mass and inflammation whereas pLPHC diet upregulates FGF21 to decrease obesity and insulin resistance. Fat mass and HOMA2-IR showed a moderate positive correlation to Proteobacteria and Desulfovibrionaceae and moderate negative correlation to the alpha-diversity (Shannon index) (Supplementary Table S3). *Akkermansia* was strongly and negatively associated with HOMA2-IR (Figure 4). Phylum Bacteroidetes showed a significant negative and positive association with HOMA2-IR (*r* = -0.58, *p* = 0.005) and alpha-diversity (Shannon index, *r* = 0.49, *p* = 0.025), respectively. In summary, this indicates that the obesity-protected, insulin-sensitive, less inflamed phenotype of pLPHC diet mice is tightly associated with higher gut bacterial diversity, increased Bacteroidetes and *Akkermansia* and lower Proteobacteria and Desulfovibrionaceae. This suggests that changes in GM may have contributed to the generally healthier phenotype of mice on the pLPHC diet.

## Discussion

We currently investigated the effect of different dietary interventions with a particular focus on pLPHC diet and concurrent activity wheel training on the GM. We found that mice on pLPHC diet had a GM profile which was overall consistent with a push of the phenotype toward a healthier state. Whether the GM profile is causally related to the host pLPHC diet phenotype, which included obesity-protection despite increased total energy intake due to increased adaptive thermogenesis and increased glucose tolerance and whole-body insulin sensitivity (Li et al., 2018a), is currently unclear. In contrast, concurrent voluntary exercise training had small and statistically insignificant effects on the GM in the current study.

An obesigenic HFD has repeatedly been reported to decrease alpha-diversity and suppress the B/F ratio whereas these parameters were normalized on pLPHC and chronic LPHC diet. Presently, Shannon-diversity was significantly higher on chronic LPHC diet than on the pLPHC regimen, as were the effect-size on the B/F ratio (pLPHC: 0.79 vs. LPHC: 1.38), suggesting a dependence on LPHC diet-duration or, equally plausible, that the repeated switching back to HFD in the pLPHC diet regimen caused an intermediate GM phenotype. It should also be noted that the 12-week pLPHC diet study ended with 14 days on LPHC diet. Thus, the phenotype may reflect mainly the impact of the last 14 days on LPHC diet rather than the chronic cumulative effect of being on pLPHC for 12 weeks. Indeed, we found this to be the case for glucose metabolism which was not different in a follow-up pLPHC study ending with 14 days of HFD, suggesting a transient improvement during each LPHC period (Li et al., 2018a). FGF21 induction, however, did show a cumulative effect and was potentiated with each additional pLPHC period, despite returning to baseline in the intertwining HFD periods (Li et al., 2018a). This might be due to epigenetic *fgf21* promotor methylation (Yuan et al., 2018) but might also be modulated by the microbiota. The HPLC diet exhibited lower Shannon-diversity in our study and had a B/F ratio of 1.96, the highest of any group. However, the Bacteroidetes and Firmicutes phyla both encompass a wide range of sub-taxa which respond differently to diet changes. In other words, the similar B/F ratio on HPLC, LPHC, and chow diet represents distinct changes in bacterial communities at lower levels of classification. This is supported by Figure 2B, which shows, e.g., a greater relative abundance of bacteria belonging to the phyla Bacteroidetes (Porphyromonadaceae, *Parabacteroides*, *Odoribacter*, *Alistipes*, *Bacteroides*, and *Alloprevotella*) in the HPLC group compared to the HFD group, whereas the bacteria belonging to the phyla Firmicutes (Lachnospiraceae, *Ruminococcus*, and *Oscillospira*) are lower in relative abundance.

Increased body weight, adiposity and decreased glucose tolerance were significantly negatively correlated to bacteria like *Akkermansia*. This may have contributed to the improved metabolic health and decreased inflammation on pLPHC diet since prebiotic feeding of *Akkermansia* was shown to reverse HFD-associated phenotypes, including obesity, endotoxemia, inflammation, and insulin resistance (Everard et al., 2013). On the other hand, the genera *Oscillibacter* and *Oscillospira*, and family Desulfovibrionaceae have all been suggested to play an active role in metabolic diseases and gut dysbiosis (Tilg and Kaser, 2011; Lam et al., 2012; Walters et al., 2014; Hamilton et al., 2015; Hu et al., 2015; Zhang-Sun et al., 2015). Altogether, the diet intervention of pLPHC seemed to positively affect several physiological parameters as well as promoting a balanced cecal microbiota. The high level of Proteobacteria detected in the HPLC group is, on the other hand, an indication of gut microbiota dysbiosis (Shin et al., 2015), which corresponds with earlier observations that HPLC has been reported to worsen metabolic outcomes (Solon-Biet et al., 2014, 2015).

Concurrent exercise training had only minor and non-significant effects on the GM and inflammatory markers despite the mice running ∼5 km/day (Z. Li, unpublished data) and showing clear exercise-training adaptations in quadriceps muscle (Li et al., 2018a). The changes included tendencies (non-significant) to higher B/F ratio on LPHC diet (non-exercise 0.79 vs. exercise: 1.25), predominantly driven by higher Bacteroidetes, and suppression of the pro-inflammatory marker IL-6. Starting with the latter, a suppressive effect of exercise training on resting IL-6 levels is well-known (Fischer, 2006). Notably, this is different from the effect of acute exercise which augments IL-6 release as a myokine from the exercising muscle (Fischer, 2006). We saw some fair correlations between gut microbial changes and IL-6 levels, positively with phylum Proteobacteria (*r* = 0.48, *p* = 0.067) and Desulfovibrionaceae (*r* = 0.48, *p* = 0.069) bacteria and negatively with the phylum Bacteroidetes (*r* = -0.51, *p* = 0.049) and the *Bacteroides* (*r* = -0.54, *p* = 0.048) genus, suggesting potential links between these. When comparing to other studies, different endurance-type exercise protocols in rodents and man have previously been shown to inconsistently influence the GM, as recently reviewed (Mach and Fuster-Botella, 2017). Studies in mice generally report higher alpha-diversity with exercise, but inconsistently report both lower and higher B/F ratio (Evans et al., 2014; Lambert et al., 2015; Denou et al., 2016 ). The GM of housed rodents is known to differ markedly between vendors and laboratories (Ericsson et al., 2015). Since the net change in bacterial communities will be dependent on the starting species composition and their competitive advantage on a given intervention, this may explain some of the differences between these studies. Interestingly, a recent mouse study ascertained the effect of voluntary activity wheel vs. forced treadmill running exercise compared to sedentary control for 8 weeks (Lamoureux et al., 2017). Similar to our study, this study reported minor and insignificant microbial profile changes when using conventional microbiome analysis. However, using a random forest machine learning model they were able to predict the voluntary exercised mice with 97% accuracy, driven by increases in both known exercise-responsive taxa [Bacteroidetes, S24-7 (Ormerod et al., 2016) and *Lactobacillus*] and previously unknown exercise-responsive taxa (Rikenellaceae and Lachnospiraceae). These taxa did not respond significantly in the current study although the average levels were generally higher with exercise, see Supplementary Figure S4. This suggests that novel non-conventional ways of analyzing the GM may more accurately describe and predict the exercise (and diet) responses and hopefully identify some consistent changes in sub-taxa predictive of health-outcomes.

We measured 92 proteins in plasma using a commercial multiplex mouse kit. Of these, 89 were detectable in plasma and we could detect significant changes in 19 proteins. Why some of these proteins, e.g., IL-23r – a transmembrane-receptor – is detectable in plasma is unclear, whereas others are more biologically feasible, e.g., Dlk1 which is known to be released as a soluble form by ADAM17-dependent cleavage (Sul, 2009). The changes in these proteins provide a potential resource for hypothesis-generation but should be validated by other methods. Currently, we focused on well-described endocrine factors, among which 8 were significantly regulated by diet and/or exercise. These included changes in 5 immune function-related proteins, among which IL-6 and Ccl3 both decreased by exercise and correlated with obesity and insulin-sensitivity in our mice, likely due to increased and suppressed inflammation by HFD and exercise, respectively. In contrast, functions for increased IL-17f by pLPHC diet and exercise, and increased Vsig2 and Ccl20 by pLPHC diet cannot be readily hypothesized based on the literature (Chretien et al., 1998; Comerford et al., 2010; Pappu et al., 2011). Follistatin was lower on pLPHC vs. HFD and also tended to be lower in exercise vs. non-exercise (*p* = 0.06). Follistatin is an endogenous inhibitor of myostatin-signaling in muscle (Sartori et al., 2014). Thus, lower follistatin might contribute to lower muscle mass in the pLPHC mice. Increased circulating ghrelin in pLPHC diet-fed mice is consistent with increased total energy intake in these mice. Neurotrophin-3 (Ntf3) was decreased by exercise training. Ntf3 is suggested to regulate survival and differentiation of mammalian neurons and may be involved in proprioception by Ia muscle afferents (Klein et al., 1994), providing a potential link to exercising muscle.

In summary, we presently compared the cecal GMs of mice on periodized LPHC diet ± voluntary exercise training against a range of chronic reference diets including regular chow, high-fat, LPHC, and HPLC diet and found that the microbiome profile on pLPHC diet was associated with health-benefits in mice similar to chronic LPHC diet, with little additional effect of exercise training.

## Data Availability

Illumina Nextseq output data is available with accession # PRJEB31608 at EMBL-EBI.

## Ethics Statement

This study was carried out in accordance with the recommendations of Declaraton of Hensinki II (1996). The protocol was approved by the Danish Animal Experiments Inspectorate.

## Author Contributions

TJ conceived and supervised the study, drafted the manuscript, and acquired funding. ZL and TJ developed the methodology. ZL, MR, JL, CHO, and TJ performed the experiments. ZL, TR, WK, and DN performed the formal analysis. ZL and TR visualized the data, and reviewed and edited the manuscript with input from all authors.

## Conflict of Interest Statement

The authors declare that the research was conducted in the absence of any commercial or financial relationships that could be construed as a potential conflict of interest.
