# Supplementary material for: The Gut Microbiome on a Periodized Low-Protein Diet Is Associated With Improved Metabolic Health
Source: Front Microbiol. 2019 Apr 4;10:709. doi: 10.3389/fmicb.2019.00709 (PMC6458274; doi:10.3389/fmicb.2019.00709)
Supplement: Supplementary file 1 [file Data_Sheet_1.docx]

Supplementary Material


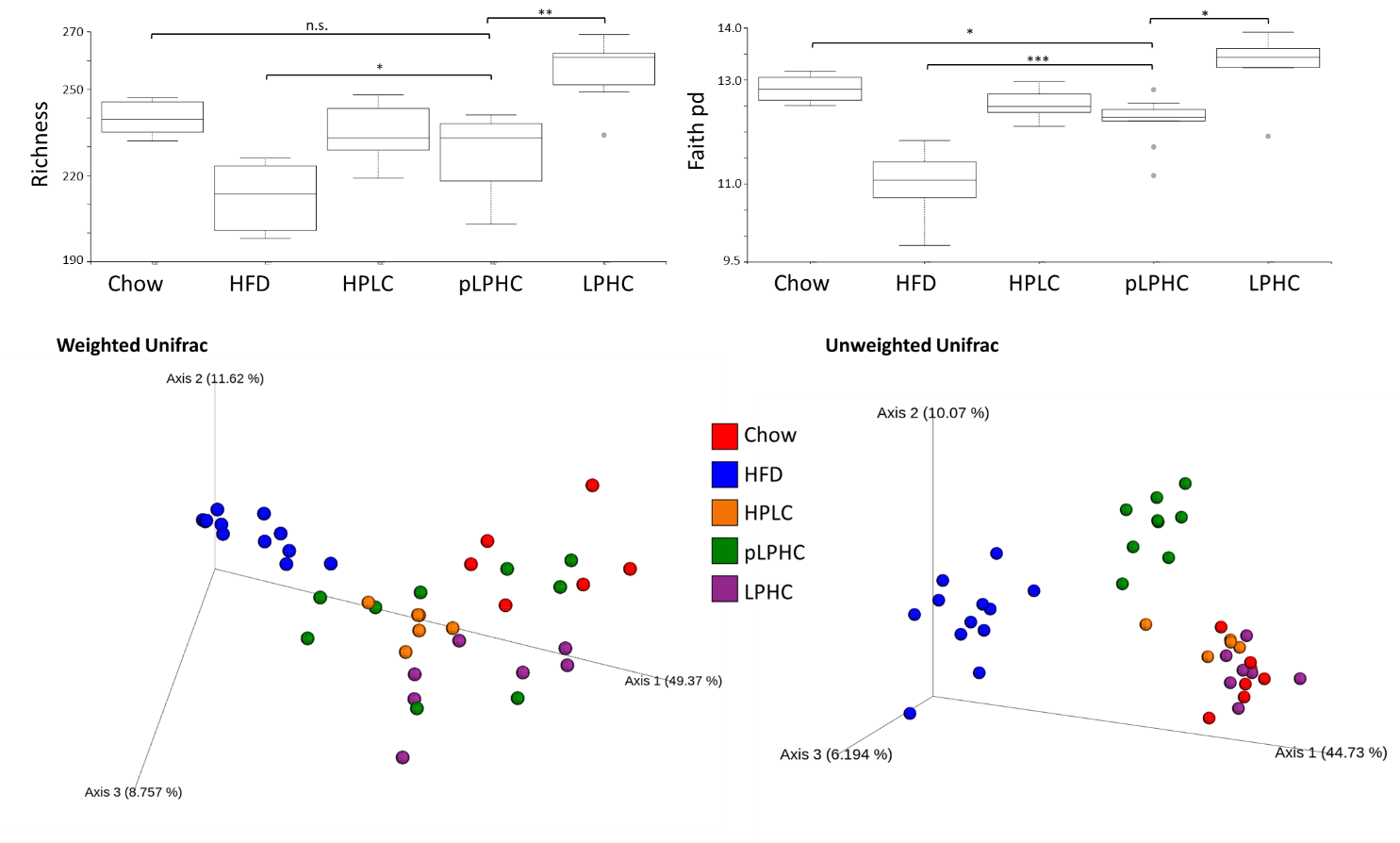


|  |  |  | **Shannon Index** | | **Richness** | | **Faith pd** | | **Bray Curtis** | | **W-Unifrac** | | **UnW-Unifrac** | |
| --- | --- | --- | --- | --- | --- | --- | --- | --- | --- | --- | --- | --- | --- | --- |
| **Gr.1** | **Gr. 2** | **n** | **H** | **p-val** | **H** | **p-val** | **H** | **p-val** | **R** | **p-val** | **R** | **p-val** | **R** | **p-val** |
| Chow | HFD | 18 | 6.8772 | 0.0087 | 11.391 | 0.0007 | 11.3684 | 0.0007 | 0.850 | 0.001 | 0.990 | 0.001 | 0.985 | 0.001 |
|  | HPLC | 12 | 5.0256 | 0.0250 | 0.7894 | 0.3743 | 3.1026 | 0.0782 | 0.727 | 0.006 | 0.638 | 0.003 | 0.451 | 0.002 |
|  | pLPHC | 15 | 2.3472 | 0.1255 | 3.1475 | 0.0760 | 7.3472 | 0.0067 | 0.476 | 0.002 | 0.243 | 0.035 | 0.818 | 0.001 |
|  | LPHC | 13 | 9.0000 | 0.0027 | 5.2389 | 0.0221 | 4.5918 | 0.0321 | 0.683 | 0.002 | 0.494 | 0.003 | 0.555 | 0.002 |
| HFD | HPLC | 18 | 0.2193 | 0.6396 | 8.4385 | 0.0037 | 11.3684 | 0.0007 | 0.518 | 0.001 | 0.923 | 0.001 | 0.967 | 0.001 |
|  | pLPHC | 21 | 12.6263 | 0.0004 | 4.2502 | 0.0392 | 11.1566 | 0.0008 | 0.929 | 0.001 | 0.759 | 0.001 | 0.853 | 0.001 |
|  | LPHC | 19 | 12.6000 | 0.0004 | 12.611 | 0.0004 | 12.6000 | 0.0004 | 0.881 | 0.001 | 0.959 | 0.002 | 0.988 | 0.001 |
| HPLC | pLPHC | 15 | 8.0000 | 0.0047 | 0.7826 | 0.3763 | 1.6806 | 0.1949 | 0.784 | 0.001 | 0.255 | 0.020 | 0.793 | 0.001 |
|  | LPHC | 13 | 9.0000 | 0.0027 | 6.6122 | 0.0101 | 4.5918 | 0.0321 | 0.742 | 0.003 | 0.502 | 0.002 | 0.711 | 0.002 |
| pLPHC | LPHC | 16 | 7.2857 | 0.0070 | 8.4734 | 0.0036 | 6.7255 | 0.0095 | 0.579 | 0.001 | 0.258 | 0.014 | 0.802 | 0.001 |

Figure S1: Additional alpha and beta-diversity metrices were applied and produced the same overall tendencies. Richness and Faith phylogenetic diversity were used in addition to Shannon index and weighted and unweighted Unifrac (W-Unifrac & UnW-Unifrac) were used in addition to the Bray-Curtis distances. All statistical pairwise group comparisons are listed in table below (Kruskal Wallis for alpha-diversity and ANOSIM for beta-diversity). Abbreviations: HFD = high fat diet, HPLC = high protein low carbohydrate, pLPHC = periodized low protein high carbohydrate, * = p < 0.05, ** = p < 0.005, *** = p < 0.0008, n.s. = not significant.


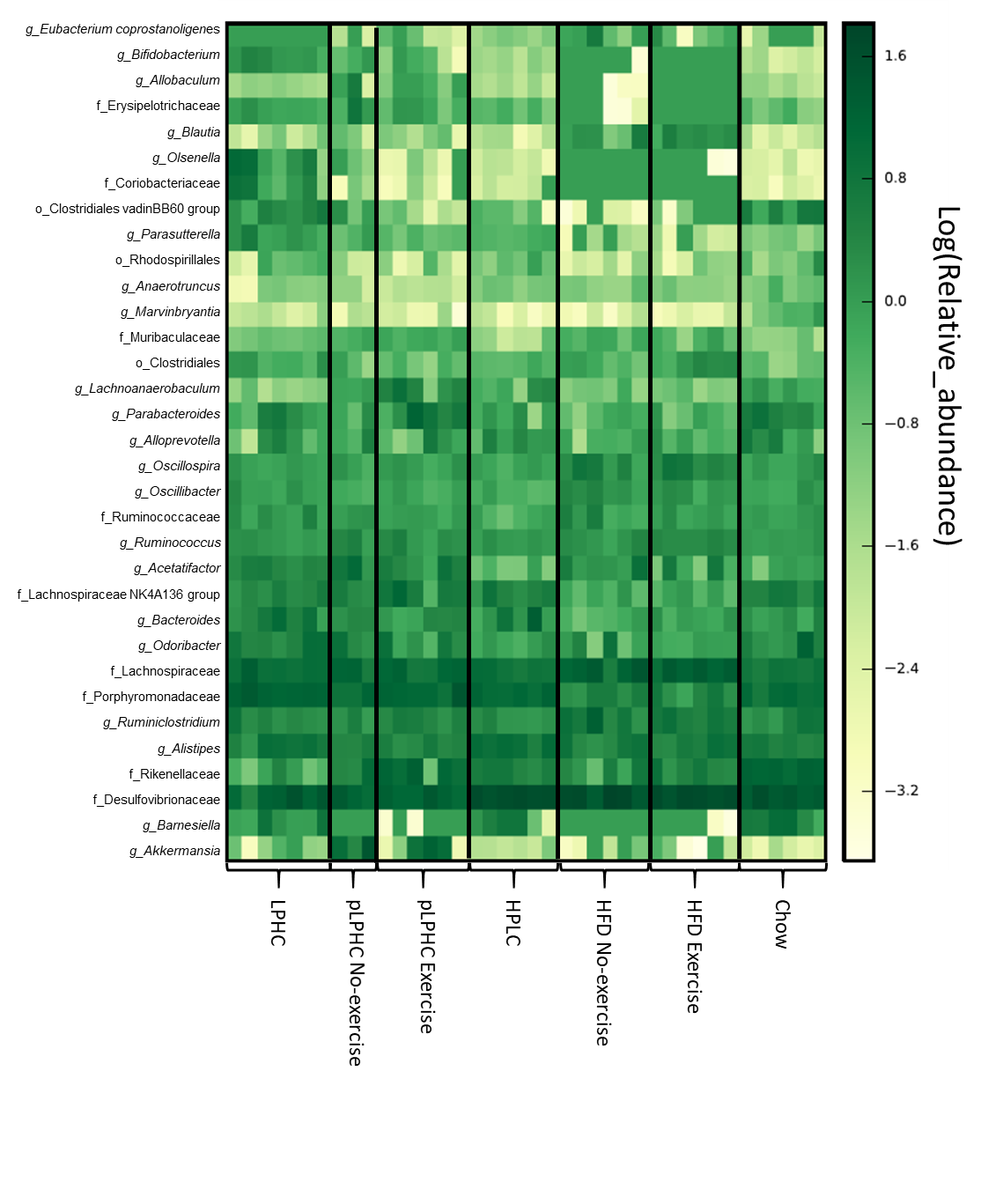
Figure S2: Heatmap showing log(relative abundance) of selected bacterial taxa on a given diet (± voluntary exercise) in the individual mice.


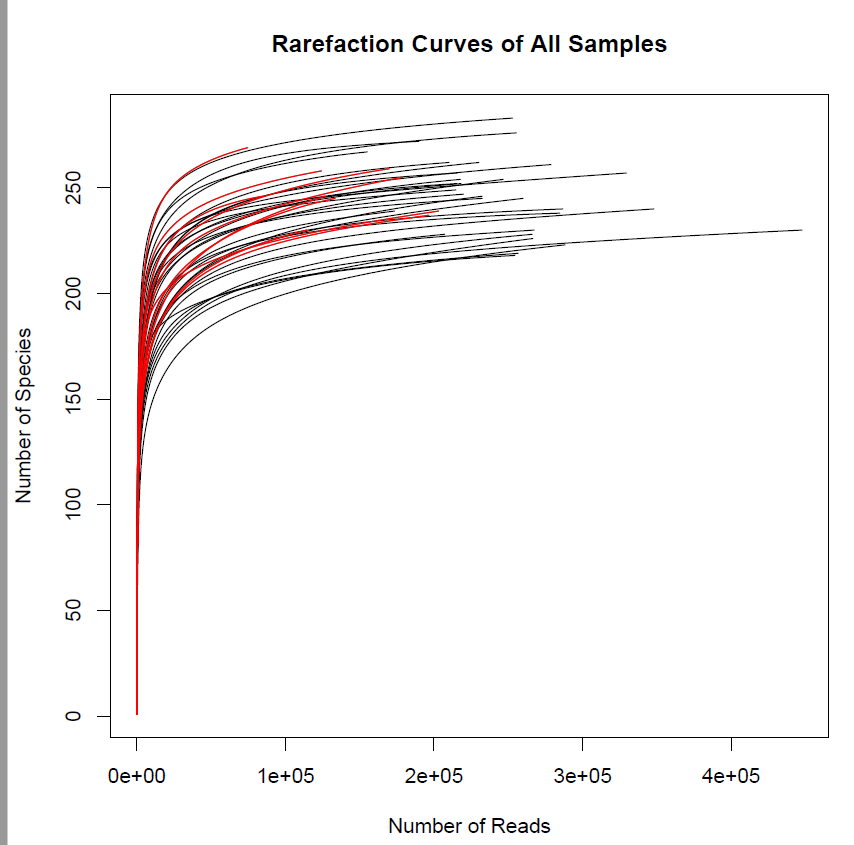


Figure S3: Rarefaction curve of all samples included in the study. The red lines indicate the samples with the steepest rarefaction curves.


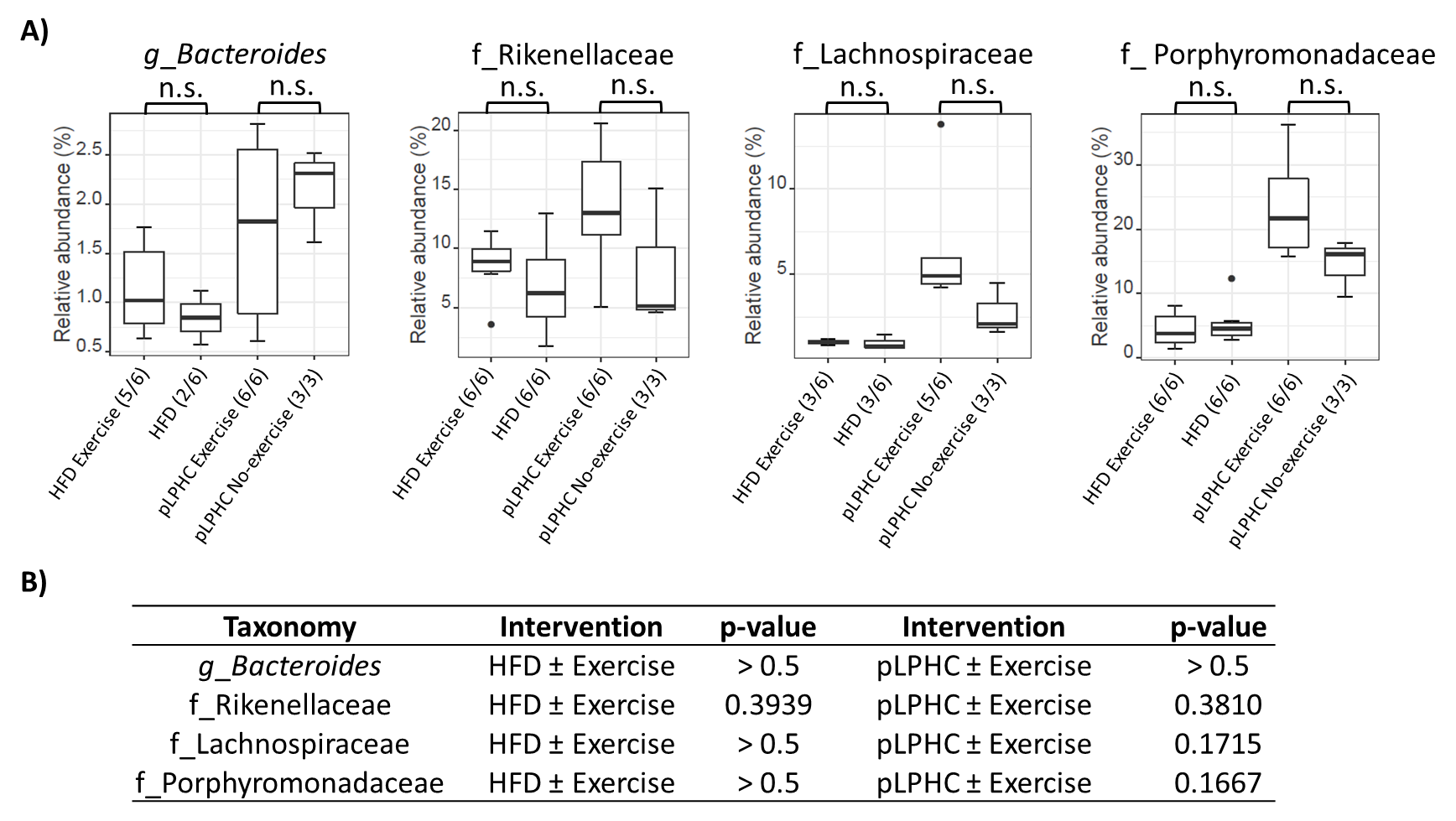
Figure S4: A) Box and whiskers plot highlighting the effect of exercise on selected bacteria taxonomy, which has been reported to increase in relative abundance due to voluntary exercise [18]. B) Table listing the significance based on pair wise Wilcoxon rank sum test. Abbreviations: n.s. = not significant, HFD = High-fat diet, pLPHC = periodized low-protein high-carbohydrate diet.

| **Gene name** | **UniProt**  **ID** | **Significance** | **Fold Change**  **InterDiet/HFD** | **P value InterDiet/HFD** | **Fold Change**  **Exercise/Non EX** | **P value Exercise/Non EX** |
| --- | --- | --- | --- | --- | --- | --- |
| Il17f | Q7TNI7 | Diet/EX | 1.84 | 0.026 | 0.56 | 0.034 |
| Vsig2 | Q9Z109 | Diet | 1.54 | 0.003 | 1.15 | 0.363 |
| Il23r | Q8K4B4 | Diet | 1.43 | 0.003 | 0.99 | 0.969 |
| Fst | P47931 | Diet | 0.69 | 0.010 | 0.76 | 0.059 |
| Ghrl | Q9EQX0 | Diet | 1.68 | 0.005 | 0.97 | 0.898 |
| Ccl20 | O89093 | Diet | 1.51 | 0.045 | 0.85 | 0.454 |
| Dlk1 | Q09163 | Diet | 1.43 | 0.000 | 0.90 | 0.339 |
| Tpp1 | O89023 | Diet | 1.34 | 0.005 | 1.03 | 0.827 |
| Cntn1 | P12960 | Diet | 1.20 | 0.019 | 1.08 | 0.333 |
| Tnr | Q8BYI9 | Diet | 1.30 | 0.035 | 0.90 | 0.404 |
| Il6 | P08505 | Ex | 0.77 | 0.167 | 0.66 | 0.041 |
| Ccl3 | P10855 | Ex | 0.80 | 0.337 | 0.62 | 0.031 |
| Ntf3 | P20181 | Ex | 0.98 | 0.895 | 0.76 | 0.016 |
| Riox2 | Q8CD15 | Ex | 0.94 | 0.594 | 0.73 | 0.006 |
| Ddah1 | Q9CWS0 | Ex | 0.97 | 0.910 | 0.58 | 0.035 |
| Dctn2 | Q99KJ8 | Ex | 0.93 | 0.728 | 0.67 | 0.040 |
| Tgfbr3 | O88393 | Ex | 1.02 | 0.729 | 0.88 | 0.015 |
| Flrt2 | Q8BLU0 | Ex | 0.94 | 0.624 | 0.79 | 0.042 |
| Apbb1ip | Q8R5A3 | Ex | 0.94 | 0.487 | 0.82 | 0.021 |

**Table S1: Significantly deregulated proteins in plasma**

| **Gene name** | **UniProt**  **ID** | **Significance** | **Fold Change**  **InterDiet/HFD** | **P value InterDiet/HFD** | **Fold Change**  **Exercise/Non EX** | **P value Exercise/Non EX** |
| --- | --- | --- | --- | --- | --- | --- |
| Il17f | Q7TNI7 | Diet/EX | 1.84 | 0.026 | 0.56 | 0.034 |
| Vsig2 | Q9Z109 | Diet | 1.54 | 0.003 | 1.15 | 0.363 |
| Il23r | Q8K4B4 | Diet | 1.43 | 0.003 | 0.99 | 0.969 |
| Fst | P47931 | Diet | 0.69 | 0.010 | 0.76 | 0.059 |
| Ghrl | Q9EQX0 | Diet | 1.68 | 0.005 | 0.97 | 0.898 |
| Ccl20 | O89093 | Diet | 1.51 | 0.045 | 0.85 | 0.454 |
| Il6 | P08505 | EX | 0.77 | 0.167 | 0.66 | 0.041 |
| Ccl3 | P10855 | EX | 0.80 | 0.337 | 0.62 | 0.031 |
| Ntf3 | P20181 | EX | 0.98 | 0.895 | 0.76 | 0.016 |
| Tnf | P06804 | None | 0.93 | 0.320 | 1.02 | 0.832 |
| Cxcl1 | P12850 | None | 0.65 | 0.053 | 0.70 | 0.108 |
| Ccl5 | P30882 | None | 0.88 | 0.605 | 0.79 | 0.326 |
| Ccl2 | P10148 | None | 0.75 | 0.265 | 0.71 | 0.187 |
| Fas | P25446 | None | 1.36 | 0.265 | 0.62 | 0.082 |
| Il1a | P01582 | None | 1.26 | 0.315 | 0.65 | 0.055 |
| Cxcl9 | P18340 | None | 0.72 | 0.249 | 0.62 | 0.089 |
| Gcg | P55095 | None | 0.95 | 0.865 | 1.11 | 0.727 |
| Gdnf | P48540 | None | 0.64 | 0.369 | 1.42 | 0.481 |
| Il17a | Q62386 | None | 1.22 | 0.563 | 0.63 | 0.171 |
| Tnfrsf11b | O08712 | None | 0.85 | 0.320 | 0.86 | 0.329 |
| Tgfb1 | P04202 | None | 0.99 | 0.885 | 1.01 | 0.910 |
| Tgfa | P48030 | None | 0.90 | 0.572 | 0.89 | 0.510 |
| Epo | P07321 | None | 0.06 | 0.276 | 4.79 | 0.421 |
| Kitlg | P20826 | None | 0.86 | 0.156 | 1.13 | 0.278 |
| Hgf | Q08048 | None | 0.96 | 0.669 | 1.12 | 0.241 |
| Il10 | P18893 | None | 0.67 | 0.151 | 0.92 | 0.772 |
| Tnfrsf12a | Q9CR75 | None | 1.06 | 0.662 | 1.13 | 0.380 |
| Il1b | P10749 | None | 0.70 | 0.057 | 0.91 | 0.657 |
| Pdgfb | P31240 | None | 1.12 | 0.166 | 1.10 | 0.256 |
| Eda2r | Q8BX35 | None | 1.04 | 0.677 | 0.99 | 0.939 |
| Tnfsf12 | O54907 | None | 0.87 | 0.341 | 0.92 | 0.576 |
| Vegfd | P97946 | None | 0.92 | 0.343 | 0.94 | 0.515 |

**Table S2: Effect of pLPHC or Exercise on plasma protein of interests**

**Table S3**: Correlations between physiological parameters and bacterial taxonomy, based on Pearson’s. α-diversity includes richness, Shannon index, and Simpson indices. Blue = positive, red = negative, n.s. = not significant, r = Pearson’s r.

|  |  | **Fat mass** | | **HOMA2-IR** | | **α-diversity** | | **Vsig2** | | **FGF21** | | **Ghrelin** | |
| --- | --- | --- | --- | --- | --- | --- | --- | --- | --- | --- | --- | --- | --- |
| **Taxonomy** | **n** | **r** | **p-val** | **r** | **p-val** | **r** | **p-val** | **r** | **p-val** | **r** | **p-val** | **r** | **p-val** |
| p_Bacteroidetes | 21 | < 0.5 | n.s. | -0.59 | 0.0054 | +0.52 | 0.0146 | < 0.5 | n.s. | < 0.5 | n.s. | < 0.5 | n.s. |
| p_Proteobacteria | 21 | +0.71 | 0.0003 | +0.55 | 0.0104 | -0.52 | 0.0146 | -0.67 | 0.0008 | < 0.5 | n.s. | -0.58 | 0.0057 |
| f_Desulfovibrionaceae | 21 | +0.71 | 0.0003 | +0.55 | 0.0094 | -0.52 | 0.0159 | < 0.5 | n.s. | -0.68 | 0.0006 | -0.59 | 0.0045 |
| f_Ruminococcaceae | 21 | < 0.5 | n.s. | +0.62 | 0.0028 | < 0.5 | n.s. | < 0.5 | n.s. | < 0.5 | n.s. | < 0.5 | n.s. |
| f_Porphyromonadaceae | 21 | -0.53 | 0.0131 | -0.57 | 0.0074 | +0.60 | 0.0042 | < 0.5 | n.s. | +0.58 | 0.0058 | +0.55 | 0.0105 |
| *g_Akkermansia* | 7 | n.s. | n.s. | -0.93 | 0.0023 | n.s. | n.s. | n.s. | n.s. | n.s. | n.s. | +0.78 | 0.0381 |
| *g_Oscillibacter* | 17 | < 0.5 | n.s. | < 0.5 | n.s. | < 0.5 | n.s. | -0.53 | 0.0305 | < 0.5 | n.s. | < 0.5 | n.s. |
| *g_Oscillospira* | 20 | < 0.5 | n.s. | < 0.5 | n.s. | < 0.5 | n.s. | < 0.5 | n.s. | -0.50 | 0.0235 | < 0.5 | n.s. |
